# Evaluation of the impact of “speak out” and “human libraries” educational methodologies on nursing students' attitudes toward immigration

**DOI:** 10.3389/fpubh.2024.1308973

**Published:** 2024-02-02

**Authors:** Vicente Gea-Caballero, Ana Pellín-Carcelén, José Luís Piera-Gomar, María Isabel Mármol-López, Iván Santolalla-Arnedo, Clara Isabel Tejada Garrido, Amaya Burgos-Esteban, María Rasal Sánchez, Amparo Rasal Sánchez, María Inmaculada Carboneres-Tafaner, Raúl Juárez-Vela

**Affiliations:** ^1^Faculty of Health Science, Research Group in Community Health and Care, Valencia International University, Valencia, Spain; ^2^Nursing School La Fe, Adscript Centre, Research Group GREIACC, Health Research Institute La Fe (IISLaFe), University of Valencia, Valencia, Spain; ^3^Predepartamental Unity of Nursing, Research Group GRUPAC, Faculty of Health Sciences, La Rioja University, Logroño, Spain; ^4^Farmamundi, Valencia, Spain; ^5^Consorcio Hospital General Universitario de Valencia, Valencia, Spain; ^6^Independent Researcher, Valencia, Spain

**Keywords:** students, nursing, emigration and immigration, attitude, non-randomized controlled trials as topic, universities

## Abstract

**Introduction:**

Measuring and understanding attitudes toward migrants is crucial in Health Sciences professionals. Nursing students, as future professionals in the healthcare system, must be comprehensively trained and prepared from the undergraduate level to effectively face the challenges of caring for health and disease processes in an increasingly globalized world. Our study aims to determine the level of attitudinal change in nursing students for immigrants, based on a training intervention with sessions of coexistence with immigrants in Spain.

**Methods:**

Quasi-experimental controlled and non-randomized study, carried out in 2019 in Nursing School La Fe, Valencia (Spain), with 201 participants (74 intervention group, 127 control group). Instrument: Attitudes toward Immigration Instrument (IAHI) questionnaire. Educational techniques of the training intervention: Speak outs and Human Libraries. Descriptive statistical analysis and comparison of results between groups was performed.

**Results:**

The participants in the intervention group showed significant changes in attitude modification, both in the total score of the questionnaire and in 4 of the 5 dimensions (pre-post intervention medition). When comparing the differences between the intervention group and the control group, we observed significant differences in 3 of the 5 dimensions: equality principles and policies, positive favorability, and negative favorability.

**Conclussion:**

Sessions involving coexistence, discussion, and reflection with immigrants, as educational intervention methods for nursing students (Speak outs and Human Libraries), are useful and effective tools to promote positive attitudinal changes toward immigrants within the healthcar context in nursing students.

## Introduction

The globalization phenomenon has led to a change in the human mobility patterns worldwide, with consistent movements of people motivated by the need and/or desire to improve their personal or family situation (1). According to INE data (Statistic National Institute of Spain), the number of foreigners in Spain increased by 183,073 people during the first half of 2019, to a total of 5,023,279 as of July 1, 2019. This increase responded, to a large extent, to a positive migratory balance of 205,678 people (2). This population growth, together with previous and massive immigration movements in the 2000-2010 decade, has led to a rapid and abrupt increase in Spain's population that affected society at all levels. The healthcare system and its professionals have also been affected, and have not been properly prepared either quantitatively or qualitatively, as the number of healthcare personnel has not increased proportionally and they have not received the necessary cross-cultural training (3).

As future professionals in the healthcare system, nursing students must be competently trained and prepared from the undergraduate level to face the challenges of caring for health and disease processes in an increasingly globalized world. Knowledge of the values, cultures, customs, and experiences of migrants is crucial for this purpose. A study carried out in 4 countries highlights that the students themselves consider their level of transcultural competence to be variable, and they agree that transcultural nursing content should be integrated in nursing study plans (4). So are crucial, the attitudes shown by the students as future professionals toward those who, coming from other places, need professional care (5). An attitude is the disposition of a person toward another person, a thing, or an idea involving three distinct components: cognitive, affective, and behavioral aspects. The cognitive component consists of the person's beliefs or knowledge about the “object;” the feelings about the “object” refer to the affective component and, finally, the behavioral component reflects a person's predisposition to act toward the “object” in a particular way (6). In our context, it means the way we think, feel and act, specifically concerning immigration (7). We will know or estimate people's attitudes by analyzing the way they speak/express themselves, their behaviors, and their ways of relating to others (8).

Measuring and knowing the attitudes toward migrants is important socially, but it is equally, if not more, important to measure them in Health Sciences professionals. In this sense, the attitudes of nursing students are important because they are a reflection of the Spanish society's attitudes toward immigration (9). Spain is well-known for its large tolerance toward immigration; however, there is a common misconception that migrants abuse the healthcare system and that this diminishes the quality of healthcare, as resources are limited. In intercultural training (a key aspect of nursing care), active training processes have shown that when trainees deeply reflect on their attitudes, they show a more receptive and global behavior toward immigrants in health care (10). Educating in values and attitudes is also a responsibility of teachers and universities since they are training future professionals. In Spain, the Nursing degree is established in the white book of the National Agency for Quality Assessment and Accreditation (ANECA) (11), which recommends addressing multiculturalism, demographic changes, and emerging health problems (among others), to achieve specific professional competencies. However, only a few Spanish universities incorporate basic subjects on transculturally, culture, or gender, being in general contents approached from basic anthropology/sociology subjects. In some universities, it is an optional subject, and in others, it is included in more global subjects that have some cultural diversity content (7).

However, this phenomenon is not something that happens exclusively in Spain. In a globalized world marked by high and increasing mobility for diverse and complex reasons, it is not surprising to find healthcare professionals with negative attitudes and behaviors toward immigrants; and for that reason, a worldwide background of studies exploring nursing students' attitudes toward immigration has been found. In general, the results obtained conclude that students' attitudes and acceptability toward caring for immigrants are positive, with little questioning of the personal/professional relationship with immigrants in need (or not in need) of professional care (12). The conclusions of a study (13) state that one of the issues that may explain the problem is that health professionals conceive immigrants as “others,” not feeling safe or competent to provide care; although it's considered that the attitudes of professionals are a fortunate factor in the problem. This study also proposes that the assessment and improvement of competence in culturally, and the promotion of training in multilanguage is interesting to improve attitudes toward immigration.

There are different tools to measure this way of thinking and acting about immigration, some of which have been used with nursing students. We highlight, due to its affinity with the focus of this study, the Attitudes Toward Immigration in Nursing Scale (EAIE) (14), developed and validated in Spain and specifically for nursing. Previous studies developed with this instrument have confirmed that there is a percentage of students with racist attitudes that can reach up to 23% of future professionals (10), no significant changes were found in nursing students following educational interventions aimed at modifying negative attitudes (15).

Knowing the realities of our students' attitudes toward migrants is important because it can help to design better intervention strategies to modulate these attitudes, avoiding behaviors of professional rejection in the health care/illness of immigrants. In this sense, education is a powerful tool to help achieve a better professional disposition for migrants with health needs. As evidenced by several studies (16, 17), this can be justified because when problems are important to some people and shared in a peer group, thereby raising awareness of the importance and seriousness of the problem, and encouraging community participation, inequalities and inequities in health are reduced (18). Therefore, the student's experience of contact with migrants can be relevant, as demonstrated by the most important theory for reducing prejudice at the interpersonal level, through intergroup contact (19). According to this theory, the best way to reduce prejudice is to have relationships between different groups.

Based on the above, our study aims to determine the level of attitudinal change in nursing students for immigrants, based on a training intervention with sessions of coexistence with immigrants in Spain.

## Methods

### Design

Quasi-experimental controlled and non-randomized study. A comparison was made between quantitative results obtained from a measurement in the intervention group after some training activities, and a similar group of students who did not receive such training intervention.

### Population and sample

The population was the nursing students of the La Fe Nursing School, Valencia (University of Valencia, Spain; total population *N* = 300). The study was conducted from February to June 2019. The sample was obtained by convenience procedure from the study population, selecting for the intervention group students of 1st and 4th year of the La Fe Nursing School. The intervention was performed in the subjects of Public Health and Community Intervention, being the inclusion criterion the willingness to participate in the study and sign the informed consent. The questionnaires were administered to a similar group of students from the same Nursing School, the control group, comprised 2nd and 3rd-year students at La Fe Nursing School in Valencia, in whom the intervention was not carried out because they did not have the subjects in which the training was given. The students in grades 1 and 4 who did not participate in the training intervention but did answer the previous questionnaire were considered the control group.

### Study variables

Independent variables: sociodemographic variables such as age, sex, country of origin, perceived social class, current academic year, participation in community associations, participation in social and community organizations (NGOs, Red Cross, Caritas, others.), and/or performance of volunteer social activities. Outcome variable: Results obtained in the Attitudes toward Immigration Instrument (IAHI) questionnaire (20), as well as the dimensions of the questionnaire and its items.

### Data collection method

Utilizing a digital questionnaire using Google Forms before and after the intervention; the instrument used was the IAHI validated for Spain (20). It is a self-administered questionnaire of 32 items, in which the subjects have to express their degree of agreement on a Likert-type scale from 1 to 5 (“I do not agree at all” to “I strongly agree”); the indexes of prejudice in the Scale of attitudes toward immigration range from 32 to 160 (the higher the score, the greater the prejudice).

The reliability (Cronbach's) is 0.90, ranging from 0.88 to 0.65 for the five factors obtained (“Principles and policies of equality:” 11 items; “Positive social distance:” five items; “Negative social distance:” six items; “Positive favorability:” five items; and “Negative favorability:” five items).

1. Principles and policies of equality: principles of equal opportunities and policies implementing egalitarian principles.
2. Positive social distance: refers to situations of close intimacy with immigrants.
3. Negative social distance: situations that would not be shared with immigrants.
4. Positive favorability: this refers to an evaluative dimension in which reference is made to the trust or quality of human relations that can be expected from dealing with immigrants.
5. Negative favorability: refers to an evaluative dimension in which reference is made to negative aspects of the immigrant's personality.

### Procedure and description of the educational techniques of the training intervention

1. Intervention design and temporal planning of its development.
2. Prior selection of the real actors for the educational techniques implementation: the project was articulated thanks to the collaboration with the non-governmental development organization farmamundi (https://farmaceuticosmundi.org), whom together with the teachers assumed the practical sessions implementation.
3. Pre-intervention measurement of the IAHI (20).
4. Carrying out the “speak-out” (21) session: speak out involves the creation of a common space where immigrants are the protagonists of their experiences. The “normal” positions of power, usually assumed by the health professional, are turned upside down. In this experience, the experts are the immigrants, who expose and comment their experiences in life and with the health system to professionals from different sectors (in our case, nursing students).
Immigrants from different cultural contexts and countries were previously selected. Participatory evaluation techniques were used, such as graphs, tables, drawings, and timelines, to focus on the different experiences lived in their origin countries and Spain after the migration process. On the other hand, specific topics for each gender were also worked on to address, in a space of trust, the gender roles that may be affecting health. The students had the opportunity to talk, ask questions, clarify doubts, and reflect on attitudes toward health during the development of both intervention techniques.
There were three groups of participants, 25, 25, and 24 (*n* = 74) students each (large group technique) who interacted with all the migrants (five migrants in each group) through brief conversations (~10 min per migrant). Subsequently, a discussion session was held in each group, with questions, especially focused on life processes and the approach to attitudinal and emotional aspects of the migrant, past experiences about health, and the role of the future health professional.
5. Carrying out the “human libraries technique” (22) session:
This technique is based on the transmission of personal life stories as a tool for social transformation and personal empowerment. The transformative process begins with the stories people told about their daily lives, creating the basis for critical dialogue and mutually trusting relationships.
Once the presentation of each migrant's life was finished, a discussion began encouraging the expression of students opinions, feelings and beliefs regarding the life experience of the main character. Conversation was encouraged about the different life situations exposed, related to the immigrant's experiences with the health system and its professionals, especially nurses and doctors.
Small groups of 6–10 participants (10 groups) were created, and each group listened for 15 min to the life of a migrant person. Finally, after listening to each life experience, the students were given the floor to express their feelings and emotions with the immigrant's life experience, and to interact with the immigrant person (23). Each migrant then changed groups so that all participants could listen to all the life stories. Finally, a large group debriefing session was held, with general reflections on the experience and the role of nurses in the process of migrants' health experiences, with freedom to participate and express ideas, experiences, feelings, or learning.
6. Post-intervention measurement of IAHI (20).

### Data analysis

Typical descriptive statistics of central tendency and dispersion (adapted to the nature of each variable) were performed internal consistency and reliability analysis were performed. A bivariate and multivariate analysis of the multiple relationships between the study variables was carried out, using appropriate statistics according to the nature of the variables. SPSS v26 data analysis software was used for this purpose.

### Ethical considerations

The study complies with European and Spanish data protection regulations (Organic Law 3/2018). Participants were informed and informed consent was given for signature. The project was approved (Code: 20199391) by the ethics and teaching research committee of the La Fe Nursing School (Valencia, Spain). The authors declared no ethical conflicts or funding of any kind. The participants did not receive any type of compensation for participating in the study.

## Results

The final total sample was 201 participants (response rate 66.7%), of whom 87.06% were women and 92.9% were born in Spain. Most of them lived in urban areas and more than 20% participated in volunteer actions, most of them declaring themselves to be of middle social class. A total of 74 students were assigned to the intervention group (1st and 4th grade students who signed the consent to participate in the training intervention activities), and a total of 127 to the control group. The intervention group showed a lower response rate than the control group (Table 1).

**Table 1:** Sociodemographic data (disaggregated by sex).

	***N***	**Mean**	**SD (standard deviation)**	**Min**	**Max**	**Men**	**Women**
Age	201	21.49254	5.502835	18	54		
	***N***	**%**	**% accumulated**				
Sex	201						
Men	25	12.44%	12.44%				
Women	176	87.56%	100%				
Country	201	%					
Spain	187	92.9				25 (12.44%)	162 (87.56%)
Bulgaria	1	0.50				0	1
Chile	1	0.50				0	1
Colombia	1	0.50				0	1
Cuba	1	0.50				0	1
Ecuador	3	1.49				0	3
Netherlands	1	0.50				0	1
Italy	1	0.50				0	1
Romania	5	2.49				0	5
Living environment							
Rural area	74	36.82				10 (4.98%)	64 (31.84%)
Urban area	127	63.18				15 (7.46%)	112 (55.72%)
Perceived social class			% acum				
Low	2	1.00	1.00			0 (0%)	2 (1%)
Medium-low	31	15.42	16.42			7 (3.48%)	24 (11.94%)
Medium	137	68.16	83.58			11 (5.47%)	126 (62.69%)
Medium-high	31	15.42	100			7 (3.48%)	24 (11.94%)
Current course of study							
1	64	31.84	31.84			8 (3.98%)	56 (27.86%)
2	46	22.89	54.73			7 (3.48%)	39 (19.4%)
3	35	17.41	72.14			6 (2.99%)	29 (14.43)
4	56	27.86	100.00			4 (1.99%)	52 (25.87%)
Otra titulación							
No	189	94.03	94.03			22 (10.95%)	166 (82.59%)
Yes	12	5.97	100.00			3 (1.49%)	9 (4.48%)
Performs social volunteering							
No	159	79.10	79.10			19 (9.45%)	140 (69.65%)
Yes	42	20.90	100.00			6 (2.99%)	36 (17.91%)

As for the differences between the intervention group and the control group before the intervention, we can see in Table 2 that, in general, there were no differences between the groups (significant differences were only observed in dimension 2, positive social distance).

**Table 2:** Preintervention differences between the two groups.

	**Individuals who did not complete treatment**		**Individuals who completed treatment**		***p*-value**
	**Mean**	**SD (standard deviation)**	**Mean**	**SD (standard deviation)**	
Equality principles and policies	40.93	6.22	41.17	5.97	0.845
Positive social distance	19.72	3.08	18.68	3.12	**0.004**
Negative social distance	10.91	2.62	11.19	2.39	0.283
Positive favorability	19.47	3.47	18.92	3.24	0.172
Negative favorability	9.34	3.2	9.69	3.04	0.341
Total	100.37	8.36	99.65	8.12	0.479

Values in bold are those showing statistical significance.

In Table 3, we can observe the results of the formative intervention in the intervention group, measured at the pre-and post-intervention level. The participants showed significant changes in attitude modification, both in the total score of the questionnaire and in 4 of the 5 dimensions (in dimension 3, the negative social distance toward migrants, the score decreased, but did not reach statistical significance).

**Table 3:** Before and after results in the intervention group.

	**Pre-intervention**		**Post Intervention**		***p*-value**
	**Intervention group**		**Intervention group**		
	**Mean**	**SD (standard deviation)**	**Mean**	**SD (standard deviation)**	
Equality principles and policies	41.17	5.97	42.47	5.95	**0.002**
Positive social distance	18.68	3.12	19.87	2.25	**0.001**
Negative social distance	11.19	2.39	11.13	2.65	0.965
Positive favorability	18.92	3.24	20.36	3.29	**< 0.001**
Negative favorability	9.69	3.04	7.83	3.13	**< 0.001**
Total	99.65	8.12	101.65	8.29	**0.005**

Values in bold are those showing statistical significance.

Finally, Table 4 shows the results attributable to the intervention, comparing the differences found between the intervention group and the control group. Although there were no significant differences in the total score, we did observe significant differences in 3 of the 5 dimensions: equality principles and policies, positive favorability, and negative favorability.

**Table 4:** Differences between the intervention and control groups post-intervention: differences attributable to the intervention.

	**Individuals who did not complete treatment**		**Individuals who completed treatment (post-treatment scores)**		***p*-value**
	**Mean**	**SD (standard deviation)**	**Mean**	**SD (standard deviation)**	
Equality principles and policies	40.93	6.22	42.47	5.95	**0.041**
Positive social distance	19.72	3.08	19.87	2.25	0.691
Negative social distance	10.91	2.62	11.13	2.65	0.284
Positive favorability	19.47	3.47	20.36	3.29	**0.024**
Negative favorability	9.34	3.2	7.83	3.13	**0.001**
Total	100.37	8.36	101.65	8.29	0.146

Values in bold are those showing statistical significance.

## Discussion

Our study aimed to determine the level of attitude change in nursing students to immigrants, following a formative intervention based on coexistence sessions with immigrants, using both large and small group work techniques. The fact that the students in the intervention and control groups did not show significant differences before the intervention in the total score of the questionnaire and 4 of the 5 dimensions, suggests that the sample had sufficient homogeneity to be confident in the results of the study (Table 2). Probably, the fact that the students were familiar with the contents of the subjects may be the explanatory factor for the fact that they had a more favorable disposition in the positive social distance and scored slightly higher. The intervention study evidences an important change of perspective toward the immigrant community after the educational intervention, so we can affirm that the results are positive. As for the intra-group differences in the pre-post intervention measurement, the results are also successful, which evidences the effectiveness of this type of activity, with changes in the total score and four of the five dimensions. Only in dimension 3, the negative social distance toward migrants, there was no statistical significance, but there was a decrease in the score. This suggests that targeted training should specifically address this is aspect to improve the score.

If we mention the differences attributable to the intervention between groups, we observe that in three of the five areas, there are significant changes (principles and policies of equality, positive favorability, and negative favorability). However, this difference does not occur when the total test score is analyzed. This coincides with other studies that show that personal contact, information about the “other group” or increased empathy, among other aspects, improve attitudes toward immigrants (24), although we may have to reflect on the fact that the intervention does not affect all dimensions equally. This is probably due to the complexity of the study phenomenon and the need for a multidimensional approach that exceeds the formative and experiential intervention carried out, of a transversal nature, requiring longer interventions over time.

This type of study is important for several reasons. Firstly, professional cultural competence is a competence linked to both personal contact and training. All this is strongly conditioned by various barriers that compromise care free of social prejudices, such as the socio-political reality of the country, cultural differences in the health-disease process, the language barrier, and even the health system itself (25). In this sense, some studies have shown that 4th year nursing students may be more prejudiced than those of other academic year, especially those of European origin and Christian religion; this fact should probably be taken with caution, but perhaps it could lead to social rejection toward other ethnic groups or religions (26, 27). However, this reality is not exclusive to Europe, since other studies in other continents (for example, in Chile) have concluded that migratory movements have also provoked the need to train nursing students in cultural competence (27). Secondly, research with experimental designs contributes to generating more robust evidence that allows the introduction of these educational methods in nursing students, but also in students from other health sciences disciplines and, why not, in professionals not trained in cultural competence.

In the areas where changes were observed, we first discuss those related to equality principles and policies, for which statistical significance was obtained. We interpret this as an indication that the group's perception of equal rights is on par with that of the host population. The items addressing this issue ask the student whether immigrants should live in neighborhoods reserved for them, whether they should have the same rights, and whether discrimination is not a serious problem in Spain. The most significant change occurs about equal rights, where in the post-test there is an increase in the number of responses that defend equality. This result is in line with studies carried out in other countries, which conclude that with better cultural competence, more egalitarian care and less inequality is achieved (28). This change is essential as it is well-established that, although the results of the research may be disparate, in general, the right to health care is very much conditioned by the implicit prejudices of the health care providers; thus observing that in effect, these prejudices affect the results of health as a public, free and universal service which is the Spanish health care system (29). Hence the important that after the intervention, the perception of discrimination toward immigrants has decreased. This is positive given that it affects even situations such as hospital childbirth processes, which are usually positive as they are not usually pathological processes, and some studies show that it is also necessary to eliminate or reduce prejudices by improving the cultural competence of the professionals (30).

Regarding the approach to the area of positive social distance, the attitudes explored focused on the importance of having immigrant colleagues or bosses, or that a group of immigrants were neighbors. The post-intervention results also show a greater acceptance of working with immigrants, whether they are colleagues at the same hierarchical level, or in the face of a vertical hierarchical difference. Likewise, acceptance of having immigrant neighbors also increases in the same direction. This point is noteworthy because there are abundant studies that confirm that the main fears of immigrant health professionals are not only idiomatic or social but also of discrimination within the workplace. This is a fact that not only affects immigrant nurses in the Spanish healthcare system but is also one of the great fears of Spanish nurses intending to migrate to other European countries (31, 32). Therefore, it is not only important to reduce the positive social distance from the Community but especially given the increasingly common possibility of having immigrant coworkers in healthcare services in a globalized world. In this sense, a study carried out in London states in its conclusions that “they are exposed to unprecedented levels of discrimination and harassment from their colleagues,” which is especially notable since this happens in countries that even have a need for health professionals who are usually host country for these professionals (33).

In the area of positive favorability, which evaluates the belief of whether immigrants are hard-working, honest, and educated, whether we would marry or have sexual relations with them, a decrease in negative responses was observed, which shows that after the group techniques applied, the perception toward the immigrant varies, understanding this experience from a global and complex point of view. Particularly important are the items focused on honesty, education, and willingness to work since these are health and healthcare environments, in which it is common to share work with immigrants. Some studies agree that this type of cooperative learning situation is especially productive in increasing the ease of assuming the emotional perspective of others, which leads to a change in attitudes (24, 34). This confirms the effectiveness of the intervention in this dimension.

In the area of negative favorability (whether immigrants are intolerant, whether they hold negative attitudes toward women, or whether they are habitually irresponsible) significant changes were also observed, and it can be affirmed that in all the questions there is a decrease in these perceptions after the intervention. This change in perspective is much more accentuated in the item referring to the attitude toward the female gender, which is especially important if we consider that the number of migratory movements of women has increased greatly in recent years (35), and that this is also a very important risk factor for gender violence (36). Moreover, this fact is also highly noteworthy given that nurses are key agents in the prevention and early detection of situations of risk of abuse in Spain (37). It is, therefore, crucial that there are no negative professional attitudes toward women, so as not to underestimate cases of risk. This area and in relation to care for women is especially relevant, since there are studies that affirm that migrant women perceive this discrimination, which is why they give up seeking medical attention, sometimes putting their health at serious risk (38).

In the area of negative social distance, no significant changes were observed after the post-test. This area does not refer to professional relationships, but more specifically to personal and social relationships, such as having friendships with immigrants, whether immigrants are a threat, or whether the state should take on language education programs for immigrants. Perhaps the generality of the dimension could be the cause of the non-existence of differences, as it may provoke socially desirable responses.

However, it is likely that interventions such as the one carried out in our study will not be sufficient. Some studies conducted in other countries have addressed this issue, with the aim of finding effective interventions that can also help to improve students' attitudes toward migrants. Thus, although the attitudes analyzed were, as in our study, acceptable and positive, the implementation of exchange and mobility programs for students (12) has been proposed as measures that have also shown positive results. Other studies recommend that nursing programs integrate educative sessions on transcultural nursing into the curricula of nursing education, assessing the multicultural competence of health professionals and also motivating students in language learning (13, 39). Other suggested interventions including the use of role play as a teaching-learning activity (as creating inclusive environments helps students better understand the vulnerabilities of at-risk groups) (40).

This study is applicable to nursing teaching, to try to achieve an education free of prejudices and stereotypes that results in more equitable health care that is respectful of Human Rights, in this case of vulnerable migrants, and reduce inequality in health care (28). In addition, achieving adequate cultural competence in care, based on the acquisition of knowledge, skills and attitudes, will allow obtaining better results for patients of all types (41, 42).

### Limitations

The study has some limitations. The sampling method, of convenience in selecting the members of the control group according to whether they took a specific subject, is the first limitation that we must consider, although the population was sufficiently homogeneous and freedom was given for participation in the study. In addition, the size of the final sample of the groups makes us consider that larger studies are necessary to obtain more robust and reliable results. Finally, the fact of observing significant differences in one of the five dimensions in the pre-intervention questionnaire measurement may have had some influence on the result, although, as we have previously discussed, it may simply be a consequence of knowing the curriculum of the subject. Additionally, we think that another limitation is that the manuscript does not discuss the results according to gender, being necessary to incorporate the gender perspective in this study phenomenon.

## Conclusions

The educational techniques used (speak out and human libraries) have been shown to be useful and effective tools to promote a positive attitudinal change toward immigrants in the context of health care and professional care in nursing students. Having nursing students share life experiences, temporary spaces, and collective work with immigrants improves their attitudes toward them in aspects such as principles and policies of equality, positive favorability, and negative favorability.

Improving the attitudes of nursing students toward immigrants is important because they are future professionals who must be prepared and develop competencies for interculturality and diversity in a globalized world.

## Data availability statement

The raw data supporting the conclusions of this article will be made available by the authors, without undue reservation.

## Ethics statement

The project was approved by the Ethics and Teaching Research Committee of the Escuela de Enfermería La Fe de Valencia. The studies were conducted in accordance with the local legislation and institutional requirements. The participants provided their written informed consent to participate in this study. Written informed consent was obtained from the individual(s) for the publication of any potentially identifiable images or data included in this article.

## Author contributions

VG-C: Conceptualization, Data curation, Formal analysis, Funding acquisition, Investigation, Methodology, Project administration, Resources, Writing—original draft, Writing—review & editing. AP-C: Investigation, Methodology, Project administration, Resources, Writing—original draft. JP-G: Investigation, Methodology, Writing—original draft, Writing—review & editing. MM-L: Project administration, Resources, Writing—original draft. IS-A: Formal analysis, Investigation, Methodology, Writing—original draft. CT: Data curation, Formal analysis, Visualization, Writing—review & editing. AB-E: Project administration, Resources, Writing—original draft. MR: Software, Supervision, Writing—original draft. AR: Validation, Visualization, Writing—original draft. MC-T: Methodology, Supervision, Writing—original draft, Writing—review & editing. RJ-V: Data curation, Formal analysis, Methodology, Resources, Supervision, Validation, Visualization, Writing—original draft, Writing—review & editing.
